# What makes humanity humane

**DOI:** 10.1186/1747-5333-1-14

**Published:** 2006-11-29

**Authors:** Karl H Pribram

**Affiliations:** 1 Cognitive Neuroscience, Department of Psychology Georgetown University, 37^th ^and O Streets, Washington, DC 20057-1001, USA

## Abstract

Scientific and popular lore have promulgated a connection between emotion and the limbic forebrain. However, there are a variety of structures that are considered limbic, and disagreement as to what is meant by "emotion". This essay traces the initial studies upon which the connection between emotion and the limbic forebrain was based and how subsequent experimental evidence led to confusion both with regard to brain systems and to the behaviors examined. In the process of sorting out the bases of the confusion the following rough outlines are sketched: 1) Motivation and emotion need to be distinguished. 2) Motivation and emotion are processed by the basal ganglia; motivation by the striatum and related structures, emotion by limbic basal ganglia: the amygdala and related structures. 3) The striatum processes activation of readiness, both behavioral and perceptual; the amygdala processes arousal, an intensive dimension that varies from interest to panic. 4) Activation of readiness deals with "what to do?" Arousal deals with novelty, with "what is it?" 5) Thus both motivation and emotion are the proactive aspects of representations, of memory: motivation, an activation of readiness; emotion, a processing of novelty, a departure from the familiar. 6) The hippocampal-cingulate circuit deals with efficiently relating emotion and motivation by establishing dispositions, attitudes. 7) The prefrontal cortex fine-tunes motivation, emotion and attitude when choices among complex or ambiguous circumstances are made.

## Introduction

Reviewing what I wrote more than three decades ago [1] makes me exclaim: It's pretty damn good. Who wrote this? I'm sure many of you have had this feeling about something that you composed some time ago.

Almost everything I wrote back in 1970 I'll vouch for today – and in some cases the earlier statements express my ideas better than I am doing currently. A case in point, one that I am going to pursue in this essay, concerns the topic "coding". I had forgotten that, what today I have been subsuming under the rubrics "complexity", "non-linear dynamics" and "chaos theory" is in fact what codes are all about.

In the original essay I failed to distinguish novelty from information. Daniel Berlyne based a good deal of his career on the commonly held idea that novelty provides information in the arts and sciences. Here I want to draw a distinction between these two concepts because I can discern different brain systems that are involved in one process that can be defined as "novelty" and another that can be defined as "information."

I am going to use the term "novel" much as we use the term to describe a literary novel. The essence of a novel is its complexity, its restructuring of the familiar. In contrast, I will use the term "information" in the restricted fashion as it was defined by Shannon and Weaver: that is, as a measure of "reduction of uncertainty" – which I will use as an alternative description to "information."

For decades I was uncomfortable about equating novelty and uncertainty reduction, because "information processing" in the Shannon and Weaver sense entails the operation of the posterior convexity of the brain while the processing of "novelty and familiarization" involves the brain's fronto-limbic formations. Resolution of my discomfort came from an experiment performed in Belgium by G. Smets [2]. Smets constructed two different sets of displays: in one set, from trial to trial, he changed the number items displayed but kept the arrangement of items essentially the same. In the other set he kept the number of items constant but changed the arrangement of the items. Human subjects were asked to say which set of displays was the more aesthetically interesting. During the testing, heart and respiratory rate, blood pressure and some simple brain electrical readings were recorded.

The results of the study were conclusive: changing the pattern, the arrangement of the items in the display was much more interesting and was accompanied by body and brain changes that are usually called "arousal" and "activation". Adding or subtracting items from the display proved to evoke little interest or body response.

Smets described his displays in information theoretic terms: *Adding and subtracting items increased or decreased the amount of uncertainty, the amount of potential information, in the displays. Changing the arrangement of the items in the display was a change from the familiar: the new arrangement was perceived as novel and interesting*.

## The issue

The issue is not as abstract and dry as it sounds. What is at stake is a specific kind of process that makes mankind all too human: feelings. Feelings, emotional and motivational, were not covered in the original "What Makes Man Human" despite my having spent a good part of my research career exploring the brain processes involved in emotions and motivations. Here I want to make up for this neglect by charting the far from straightforward course of the experiments on attention that lead to a realization of the import of those results for understanding emotional and motivational feelings.

My research, as had the research of many others, ran into a puzzle: Whenever an attempt was made to use body responses to measure an emotional process, the results told us little if anything about emotion but a great deal about attention. Don Lindsley, on the basis of his work on the reticular activating core of the brain stem, had proposed an activation theory of emotion in which emotion was conceived as an overall undifferentiated attentive conscious process [3]. When the reticular core of the brain stem was destroyed animals and humans survived but in a vegetative state of "akinetic mutism". The experimental results obtained by Smets on studying the reaction to changes in pattern had little in common with Lindsley's activation process: Rather, these experiments targeted attention to specific aspects of experience once general activation had been achieved.

I was doing experiments on humans and non-human primates that paralleled those performed by Smets. My experiments were stimulated by the work of Eugene Sokolov who showed that such body and brain responses indicated that a representation, a "neuronal model," of the pattern of stimulation was constructed during familiarization [4]. When monkeys or people were exposed to visual or auditory displays in which a change in pattern of the display was made, body responses identical to those measured by Smets (changes in heart and respiratory rate and blood pressure) were activated. These responses waned with repetition; as the patterns became familiar the responses habituated.

Earlier, in an exploration of the idea that body responses are integral to feelings of emotion and motivation, my research had shown, in both human and non-human primates, that electrical stimulation of a stretch of cortex along the medial and basal surface of the brain cortex resulted in changes in blood pressure, heart and respiratory rate, gastrointestinal motility and some gross head and eye movements. I labeled this cortex the mediobasal motor cortex to distinguish it from the classical motor cortex on the lateral surface of the brain. Today we would call it the limbic motor cortex.

The basal part of this limbic motor cortex is critically connected to the amygdala, a basal ganglion shaped like an almond, located at, and within, the tip of the temporal lobe of the brain.

The results of these earlier experiments that discovered the limbic motor cortex made me wonder whether the Sokolov responses to changes in patterns of stimuli depended on the occurrence of body responses. I therefore removed the amygdala on both sides of the monkeys' brains to see what would happen to body responses [5,6]. To my surprise there was no change in body responses: *the body responses occurred just as they had before surgery – and, moreover, they persisted with repetition of the stimulus. After the amygdalectomy, the body responses did not habituate*.

Habituation, familiarization was thus shown to be dependent on the presence of an intact amygdala, a system able to process body responses. The amygdala was not involved in the production of those body responses, nor, in other experiments, was the threshold to pain altered. Rather the amygdala was shown to be important to processing an experience. An experience consisted of an exposure to a change in a pattern that had become familiar. The experience may have been painful or potentially painful; or pleasant or potentially pleasant.

In some experiments we used Pavlovian classical conditioning techniques and showed that amygdalectomized monkeys failed to become conditioned [7]. This was due on the part of the amygdalectomized monkeys – when compared to control monkeys – to have a narrowed window of response to the unconditioned stimulus so that a conjunction with the conditional stimulus failed to occur.

The current emphasis on the role of the amygdala in the processing of fear is understandable but addresses only one facet of processing by the amygdala system. Fear is not an expression of an experience of pain but a memory based anticipation of pain that may be realistic or imagined.

*Since William James, scientists and philosophers have suggested that emotional experiences and expressions per se, are accompanied by – and even initiated by – body responses. But the results of my experiments indicate that body responses help a person attain, via the amygdala, a certain kind of memory, and that emotion is due to a challenge to the pattern of that memory, not the body responses themselves. When we experience a stomach ache we do not identify that experience to be an emotion*.

## How it all began: origins of confusion

My interest in the amygdala and emotions stemmed from the experimental finding reported by Heinrich Klüver and Paul Bucy of marked taming of monkeys produced by excision of their temporal lobes [8]. (I was Bucy's first resident when he began his private practice of neurosurgery). I undertook a program of research to find out which structure of the temporal lobe was responsible for producing the taming. The program also sought to find out which of the other symptoms produced by the temporal lobectomy were produced by which part of the lobe. I found that not only taming but also other "basic instincts" – the 4 Fs: fighting, fleeing, feeding and sex – were dramatically altered by removal of the amygdala, though not by removal of any other part of the temporal lobe. A further series of experiments, some of which were reviewed above, showed that the changes in the expression of basic instincts were due to changes in an animals' processing of familiarity.

Those experimental results were complemented by electrical stimulations. Electrical stimulation of the amygdala of animals and people has produced, in ascending order of the amount of stimulation (applied randomly), reactions that range from momentary to prolonged interest; to approaching conspecifics as in sexual mounting or flirting; to retreating from the environment; to outbursts of attack, labeled "sham rage".

In short, although processing the content of familiarity is highly specific, the response engendered varies along an intensive dimension reaching from interest to rage. There are, of course, many more experimental results that flesh out the skeleton described in the above review. But this is enough to provide a kernel to the popularly accepted idea that the limbic systems contain "centers" for regulating emotions such as fear – the "fleeing" of the 4 Fs.

But the popular view has built in some difficulties. The amygdala is only one structure among those that are loosely thought of as the limbic forebrain. In fact, originally the amygdala was not included at all in the limbic circuitry. Rather, two sources converged to be baptized by Paul MacLean as the "limbic systems." One source was Paul Broca who discerned a rim of cortex around the internal edge of the cerebral hemisphere to be different from the rest of the cerebral mantle. This cortex appeared paler and was later shown to be composed of only three layers and labeled "allo (other) cortex" to distinguish it from the six layered "eu (true) cortex" that makes up most of the cerebral mantle. Broca called this rim of cortex "Le Grand Lobe Limbique."

The other source, described by J.W. Papez, consisted of the hippocampus, its downstream connections through the septal region to the mammillary bodies of the hypothalamus, thence to the anterior nuclei of the thalamus that project to the cortex of the cingulate gyrus which had been labeled the "limbic" cortex. The cingulate cortex in turn feeds back into the hippocampus. Papez reasoned that emotions tend to go round and round and this anatomical circuit does just that [9]. MacLean's limbic system encompassed Broca's cortex and Papez's circuitry and originally did not include the amygdala. It was not until MacLean joined my project in John Fulton's department at Yale that it became imperative on the basis of my results to include the amygdala in any circuitry that presumably organizes our emotions.

I had summarized my early experimental results in a paper written with one of my students, Larry Kruger, titled "Functions of the Olfactory Brain" for which we received more than 2000 reprint requests [10]. In this paper we noted that Broca's limbic three-layered allocortex had, for anatomical reasons, to be expanded in primates to include adjacent new six layered cortex. Filimonov, a Russian neuroanatomist whom I went to visit in Moscow, had labeled these new six layered accretions "juxtallocortex" (next to the allocortex). My electrical and chemical stimulations as well as the results of my behavioral experiments supported the unity in function of these old and new primate cortical zones. The most clear-cut of these zones surrounded the amygdala which had, therefore, to be included in Broca's, if not in Papez's Limbic brain.

*The stage was set for the confusion that has occurred between actual experimental results and the popular and much of the scientific understanding of the relation between emotion and the limbic systems. Experiments and anatomical analyses showed the limbic brain to be not just an old brain but in primates to contain neocortical zones. Furthermore, the functions of the amygdala system proved to have more to do with attention and the formation of memory than with emotion. This confusion is multiplied by the ambiguity of the use of the term "feeling" in English. Most often "feeling" and "emotion" are used synonymously. At other times, especially by scientists, feeling is used as the subjective aspect of emotion **and motivation*.

## Stop and go

The confusion can be resolved and in the resolution new insights are gained. We have already noted that stimulations and damage to the amygdala entail familiarization of an experience. Familiarization leads to a memory that is challenged by a novel, a change in a familiar, pattern. The challenge elicits a response that varies in intensity according to the properties of the novel stimulus.

Challenge of the familiar interrupts ongoing behavior. The interruption has been described as a "What is it?" reaction. This initial response to novelty is most often followed by a "What to do?" reaction. "What to do?" engages basal ganglia other than the amygdala: the corpus striatum composed of the caudate nucleus and the putamen. Nico Spinelli and I found that electrical stimulation of the caudate nucleus and of the putamen changes recovery cycles of event related potentials and inhibitory surrounds of receptive fields in the visual system [11]. These basal ganglia are thus engaged not only in regulating postural set as is well known, but also in changing "settings" of sensory inputs in processing "what to do".

William James contrasted emotions that "stop at the skin" with instinct, that is motivations, that "get into practical relations with the environment". Getting into practical relations entails readiness and behavioral involvement, the operations regulated by the striatum, the non-limbic basal ganglia.

Thus when we talk of feelings in the sense that they encompass both emotions and motivations, we need to keep in mind that basal ganglia other than limbic are of concern. Were we to do this, one area of confusion would become clarified.

The experimental results reviewed above can be encapsulated by stating that the amygdala is part of a stop system that processes interruptions of ongoing behavior and experience; that the basal ganglia of the corpus striatum are part of a go system that readies the organism to effectively initiate or continue involvement in behaving and experiencing. The conclusions with respect to go processing are borne out by patients with Parkinson's disease who have difficulty in starting and maintaining their interests and their behavior.

With regard to stop processing, experimental results show that persons, monkeys and dogs whose amygdala have been removed fail to establish bounds on their ongoing behavior. Amygdalectomized animals fail to respect ordinary familiar territorial boundaries on their sexual behavior and treat every social encounter as novel, failing to heed interactions that had become familiar.

We obtained the most detailed evidence of how failure to heed bounds on behavior in experiments and observations of eating. Amygdalized dogs, monkeys and people do not eat more vigorously than controls after being deprived of food, or when given greater incentive to eat, but whenever food is available they fail to curtail eating and persist longer in feeding.

Anatomically the amygdala is heavily connected by way of two large pathways with the ventromedial "nucleus" of the hypothalamus, a region that has been identified as regulating satiety. When the ventromedial nucleus is destroyed animals eat and eat and eat. Their "stop" process has been removed. Interestingly, the same sequence of responses as is observed for the amygdala is observed with electrical stimulation of this hypothalamic nucleus. Thus an intensive dimension, regulated by the brain, ranges from satiety (boredom?), through interest, approach, avoidance, and to explosive attack. This dimension is truly what we can identify as emotion.

At the hypothalamic level a "go" process has also been identified in what is called its far lateral region. Lesions of this region produce animals that won't eat at all – and will starve. In this location there is no hypothalamic nucleus, but there are tracts leading from the substantia nigra to the striatal basal ganglia. These tracts are dopaminergic and are involved in the production of Parkinson's disease.

Microelectrode experiments have shown that activity in the ventromedial and far lateral hypothalamic regions is reciprocal.

A patient who had sustained an amygdalectomy illustrates this syndrome. She had gained some 50 lbs. in the six months since surgery. Her nursing records showed that she chewed and ate whatever was before her including paper and pencils. I was examining her shortly before noon and asked her why she put things in her mouth – whether she remained inordinately hungry. She answered a definite no. I then asked if she had any specific appetites such as steak or chocolate. Again a definite no. I had so hoped that this talking primate could tell me what my non-talking monkeys could not.

I opened the door of the examining room that fortunately for my inquiry led to the common room where lunch was being served. The patient slowly made her way to the table, but when she got there she pushed other patients aside, and grabbed food with both hands and began to stuff herself. I brought her back to the examining room and asked why she had behaved the way she did. She could not tell me. As a behaviorist, I learned an important lesson which most ordinary people already know: Human behavior is not necessarily a reliable guide to human subjective experience. In the epilogue of our 1960 book *Plans and the Structure of Behavior *[12], George Miller, Eugene Galanter and I called ourselves "Subjective Behaviorists", an oxymoron at the time.

*The experimental results that I have reviewed show that certain brain systems, centered on the amygdala, regulate an intensive dimension of responses to external or body stimulation, a dimension of response that we can identify as emotion. These responses are bounded by what has become familiar. Another set of responses involving the caudate and putamen, process motivated behaviors that bring the animal or person into practical relations with the environment*.

*Both emotional and motivational systems of the brain have been shown to influence sensory processes. This influence accounts for their subjective, feeling aspects*.

## The hippocampal circuit

The experimental results reviewed should resolve much of the current confusion that is glossed over by simply relating "emotions" to "the limbic system". But what about the Papez hippocampal-cingulate circuit? Just as in the case of research on the amygdala, almost all research on damage to the hippocampus has devolved on the finding of a very specific type of memory loss: *the memory of what is happening to the patient personally fails to become familiar*. Again it is memory, and familiarity, not the processing of information, or for that matter of emotion, that is involved.

However, the processing of what is familiar is different for the hippocampal system than for the amygdala system. A series of experiments in my laboratory demonstrated that while the amygdala is processing what is novel during habituation, the hippocampus is processing the context within which habituation is happening: the hippocampus is processing what is already familiar. In a learning task, when a monkey is choosing between a "plus" and a "minus" and choosing the "plus" is always rewarded irrespective of where it is placed, *the amygdala is processing **choosing* the "plus", *the hippocampus is processing **not-choosing* the "minus", the non-rewarded cue. This was a surprising result for our group until we realized that in order to walk through a door we must process the walls so as to not bump into them. Our experience with familiar walls has become, for the moment, irrelevant, no longer "elevated" in attention. We no longer are interested in walls unless there occurs an earthquake in which instance our experience of the walls promptly dis-habituates and re-elevates into attention. The memory of walls and of the minus cue does not disappear during habituation and learning.

From all these results we could almost be ready to dismiss the hippocampal-cingulate circuit from the "limbic systems process emotions" idea. Almost but not quite: In one experiment I reversed what had been the rewarded cue after criterion performance had been reached – the reward was switched from "plus" to "minus." Monkeys with hippocampal removals had no problem in reversing their performance but once they reached a 50% level of reward they were stuck at that level for weeks [13]. It seemed that they were insufficiently motivated to try for a more substantial level of reward. After such a period of 50% performance, the monkeys took off and completed the task to a 90% performance level. The slopes of achieving 50% and 90% were normal; only that long period in the middle was different. My interpretation was that at the 50% level of reward the new build-up of "not-choosing" had not proceeded sufficiently to counter the context of "not-choosing" from the pre-reversal stage of the experiment to kick the monkey into a more productive motivational phase. Support for this interpretation came from the finding that over progressive reversals the mid-plateau of 50% performance became progressively shorter.

In another experiment, monkeys whose cingulate cortex I had removed showed a dramatic shortening of the duration of their frustration to a sudden cessation of expected rewards [14]. Frustration occurs when an always-rewarded situation becomes suddenly an always unrewarded one. The "unrewarded" context is suddenly overwhelmed with a new pattern. This is more like a change in the intensive dimension that characterizes emotion than in a change in motivation.

But there is more. The hippocampus processes hormones that organize the pituitary regulation of adrenal cortical hormones which in turn are processed by the hippocampus. Thus the anatomy and physiology of the adrenal gland is reflected in the anatomy and physiology of the amygdala circuit and the hippocampal circuit: Adrenalin (epinephrine) secreted by the adrenal core is processed by the amygdala system; cortisone secreted by adrenal cortex is processed by the hippocampal system.

Adrenal cortical hormones have a great deal to do with motivation. Treatment with these hormones, which are closely related to sex hormones, produces a feeling of euphoria, of gung-ho to do things. However, prolonged excretion of adrenal cortical hormones that occurs as a result of undue stress produces ennui and depression. The effect of hippocampal processing on motivation thus provides the context of specific patterns that are currently "irrelevant" but expresses the motivation along an intensive dimension, much as does the processing of emotions by the amygdala.

Two different patterns of electrical activity have been recorded from the hippocampus. One patterns occurs when the animal alerts to a stimulus, when "what is it"? is being processed. The other pattern is present when an animal is exploring its environment, when "what to" do might be or might become of concern [15].

*Taken together these experiments indicate that the hippocampal circuit brings together emotion: the processing of familiarity; and motivation: the processing of readiness to engage the world in a practical manner*.

## Attitude

There is a final step in this hegira regarding the limbic systems and emotions. I had been attracted to Sigmund Freud's *Project for a Scientific Psychology *in part because in the *Project *Freud developed a theory of motivation based on memory rather than on drives. Freud noted that *motivations are the prospective aspects of memories*. As described in Chapter 2 of *Freud's Project Reassessed*, Freud located motivation in the basal ganglia and drew a diagram of what today we would call a neural network [16].

In writing up my comments on the functions of the amygdala, their role in familiarization, a memory process, I suddenly realized that we could conceive of *emotions as the prospective aspects of familiarization much as Freud had conceived of motivation as the prospective aspects of memories*.

Attitudes are prospective feelings. Motivational attitudes entail striatal processing; emotional attitudes entail amygdala processes and efficiently combining motivational and emotional attitudes entails processing by the hippocampal circuit.

Attitudes are dispositional states that embody the experience of the individual (in compressed form). Attitudes are formed and reformed (reset) by structuring redundancy: each level of repetition is re-presented at the next level by a single symbol – and this process continues until all repetitions have been accounted for. In short, attitudes embody complexity, attitudes embody codes. In chapter 9 of *Brain and Perception *I have reviewed preliminary evidence of how the hippocampal circuit is involved in the construction of codes [17]. More recently, J. McClelland has created a model that shows how isocortical input becomes processed by the hippocampus, is then forwarded back to the isocortex to be interleaved into ongoing processing. In an interchange (1988) between McClelland and myself I indicated that the return to the isocortex needs to proceed via the basal ganglia [18].

In the presence of ambiguity, when choices are less than straightforward, attitudes come under the control of divisions of the frontal cortex, one division monitoring amygdala, another monitoring striatal and still another, hippocampal circuits.

Recently much attention has been paid to the involvement of emotional processes in cognitive functions [e.g., [19]]. An excellent example is a recent report in Science [20] that describes the *selective activation of the amygdala*, shown by functional Magnetic Resonance Imaging (fMRI), in what Kahneman and Tversky have called a "framing effect" [21]. The frame is, of course, the context of "what isn't" that I have described in this essay. The Science report therefore supports what I described earlier, on the basis of my experimental results, as "placing bounds, boundaries" on experience and behavior. The report also supports the conclusion that divisions of the frontal cortex become activated in modulating the framing effect.

*As my brief review indicates, when brain processes are considered, the involvement of **feelings** with reasoning and choice is much more intimate and intricate than we had suspected: "What Makes Man Human" is the humane-ness of his brain*.

## Suggested readings

I have chosen references 22–29 [22-29] as seminal to the themes covered in this essay. References 22–26 [22-26] are early studies that have become almost totally lost to current thinking about emotion and motivation. References 27–29 [27-29] are papers that have contributed and are contributing important insights to our understanding of What Makes Humanity Humane.
